# The Receding Year

**Published:** 1877-12-20

**Authors:** 


### THE RECEDING YEAR.

There is a pleasure peculiarly its own in contemplating antithetical positions. If an individual had no past, with its varied histories, its weal and woe, defeats and triumphs, sunlight and gloom, the horoscope of the present would but faintly shadow the prophecies of the future. As we round the tide of each succeeding year, and glance back at the foam-capped billows we have breasted, or the serene waters over which we have peacefully sailed, we are prone to judge of the sea before us by that over which we have passed, only that Hope, ever on her buoyant wing, and hovering near, like some silvery-breasted bird, sings her syren song for the coming year, until the heart is fain to believe that the next annual revolution of time will be more richly fruited with joy and success than the one so rapidly passing away. This is well. If this song were not sung for us as the cycles go by, vigor, ambition and perseverance would fail, and our moral, mental and physical faculties lose that buoyant action so necessary to meet, successfully, the coming events that cast both shadow and sunshine before.

Under the beneficent influence of these emotions, we turn from the pages of the last volume of the Record, and look with expectant glances into its future for the period of 1878, and with a hopeful desire to see the profession of medicine "lead the van in modern research" and progression. We hope to see it frankly accept and promulgate still broader views in medical science, even though it should operate against its own pecuniary interests for a season, among the prejudiced and uninformed.

It requires a grandeur of moral courage to drop the worn-out "husks of theories and remedies that have fulfilled their day," and are supported by the faith and the purse of patrons, for the more modern one, based upon an earnest and intelligent investigation of both psychical and physical facts, that must lay the foundation of a nobler medical science. But this is necessary to the continual and perfect development of the human race, and there should not be a more advanced or grander progressionist among men than the practitioner of medicine.

To our *confreres* in the profession, and our patrons at large, we would again proffer our warmest thanks for their support, kindly courtesy, and encouraging words during the past twelve months, and express the hope that they will generously continue their patronage and friendly interest for the year that will soon dawn upon us. Dr. Word will continue in charge of the business department, and we trust our friends will not only aid him in procuring subscriptions for the RECORD, but will continue to aid us in every department. We desire brief communications and practical items.

To each and all of our readers, and every member of our noble and heaven-born profession, we would tender the compliment of a merry Christmas, and a happy New Year.

